# Effect of *Inonotus obliquus* Extract Supplementation on Endurance Exercise and Energy-Consuming Processes through Lipid Transport in Mice

**DOI:** 10.3390/nu14235007

**Published:** 2022-11-25

**Authors:** Yi-Ming Chen, Wan-Chun Chiu, Yen-Shuo Chiu

**Affiliations:** 1 Department of Food Science, Fu Jen Catholic University, New Taipei City 242062, Taiwan; 2 School of Nutrition and Health Sciences, College of Nutrition, Taipei Medical University, Taipei City 11031, Taiwan; 3 Research Center of Geriatric Nutrition, College of Nutrition, Taipei Medical University, Taipei City 11031, Taiwan; 4 Department of Nutrition, Wan Fang Hospital, Taipei Medical University, Taipei City 11696, Taiwan; 5 Department of Orthopedics, Shuang Ho Hospital, Taipei Medical University, Taipei City 23561, Taiwan

**Keywords:** *Inonotus obliquus*, exercise performance, energy expenditure, PPAR signaling pathway, lipid transport

## Abstract

*Inonotus obliquus* (IO) is used as functional food to treat diabetes. This study investigated the effect of IO supplementation on body composition in relation to changes in energy expenditure and exercise performance. Male Institute of Cancer Research mice were divided into four groups (*n* = 8 per group) and orally administered IO once daily for 6 wk at 0 (vehicle), 824 (IO-1×), 1648 (IO-2×), and 2472 mg/kg (IO-3×). IO supplementation increased muscle volume, exhaustive treadmill time, and glycogen storage in mice. Serum free fatty acid levels after acute exercise improved in the IO supplementation group, which exhibited changes in energy expenditure through the peroxisome proliferator-activated receptor (PPAR) pathway. RNA sequencing revealed significantly increased PPAR signaling; phenylalanine, ascorbate, aldarate, and cholesterol metabolism; chemical carcinogenesis; and ergosterol biosynthesis in the IO group compared with the vehicle group. Thus, IO supplements as nutraceuticals have a positive effect on lipid transport and exercise performance. In addition, this study was only IO supplementation without training-related procedures.

## 1. Introduction

*Inonotus obliquus* (IO), also known as chaga, belongs to *Profundidae* genus of the *Poriaceae* family [1]. It is mainly distributed in latitudes between 45° and 50° N, such as Russia (Siberia), North America, Japan, and China (Heilongjiang); IO is a medicinal, edible fungus [2]. Studies have revealed that the polyphenoloxicarbonic complex of IO contains two to three types of polymer structures of different molecular mobilities. IO water extracts vary slightly according to the combination of compounds, such as phenolic substances [3], polysaccharides [4], steroid substances [5], nonsaturated fatty acids, vitamin K, coenzyme Q, phospholipids, and glycolipids. The ethyl acetate extract of IO contains phenol-carbonic acids, simple phenols, flavonoids, iridoids, and triterpenes [6]. IO has many pharmacological effects and has been demonstrated to alleviate hyperglycemia, hyperinsulinemia, and impaired glucose tolerance in mice with type 2 diabetes mellitus [7]. Its insulin-stimulated glucose uptake is directly linked to the phosphoinositide 3-kinase pathway, which increases glucose uptake through the translocation of the glucose transporter type 4 protein and suppresses interleukin (IL)-2 and IL-2R levels through the inhibition of the NF-κB protein complex in mice with streptozotocin (STZ)-induced diabetes [8]. IO also affects peroxisome proliferator-activated receptor (PPAR)-γ and sterol regulatory element-binding transcription factor 1 (SREBP1-c) transcription factors, which play critical roles in lipogenesis, changing lipid metabolism [9]. Thus, IO, as a PPAR-activating agent, can activate PPAR. PPAR is a heterotrimeric complex with a key role in stimulating energy-generating processes, such as glucose uptake and fatty acid oxidation, and decreasing energy-consuming processes, such as protein and lipid synthesis [10]. Previous studies have found out that IO supplementation can result in faster glucose transport from the blood stream in the skeletal muscle as well as its potential role to increase mitochondrial lipid oxidation which could potentially delay the lactate threshold and ultimately results in better endurance performance [11,12,13]. Therefore, IO decrease lipid accumulation is most likely due to alleviate fatty acid uptake into the muscle coupled with enhance mitochondrial lipid oxidation [14], and increase the potential of muscle growth by enhancing lipid-mediated insulin sensitization.

IO contains betulin, betulinic acid, inotodiol, and trametenolic acid, which are major triterpenoids [15]. Studies have revealed that ergostane-type triterpenoids are active antifatigue components [16]. Fatigue is an extremely complex physiological change process, generally referring to a decline in physical and mental states that make it difficult to maintain the physiological function of the body in a steady state [17]. Therefore, IO is an antifatigue herbal supplement candidate. This study explored the effect of IO supplementation on exercise performance and energy-consuming processes and identified the possible mechanism through which IO can increase exercise performance.

## 2. Materials and Methods

### 2.1. Animals and Experiment Design

The IO full-spectrum mushroom extract was purchased from Jilin Ruikang Biotechnology (Jilin, China). Male Institute of Cancer Research (ICR) mice (6-wk old), grown under specific pathogen-free conditions, were purchased from LASCO Biotechnology (Taipei, Taiwan). All the mice had free access to a standard laboratory diet (No. 5001; PMI Nutrition International, Brentwood, MO, USA) and distilled water and were housed in a 12 h light and 12 h dark cycle at room temperature (22 ± 1 °C) and 30–40% humidity. The animal protocol (LAC-2019-0459) was reviewed and approved by the Institutional Animal Care and Use Committee of Taipei Medical University, Taipei city, Taiwan. The 1× dose of IO used for humans is typically 4 g per day. The 1× mouse dose (824 mg/kg) used in this study was converted from a human-equivalent dose (HED) based on the body surface area according to the US Food and Drug Administration formula: assuming a human weight of 60 kg, the HED for 2000 (mg)/60 (kg) = 25 × 12.3 = 824 mg/kg; the conversion coefficient 12.3 is used to account for differences in the body surface area between mice and humans, as previously described. An *a priori* power analysis (G*Power version 3.1.9.4; Heinrich Heine University Düsseldorf, Düsseldorf, Germany) showed that a minimum of 8 mice was required on the basis of conventional α (0.05) and β (0.80) values, and Cohen’s d value was 1.313. The sample size *n* = 8 was enough as reported in Chen et al. [18].

In total, 32 mice were randomly assigned to four groups (8 mice/group) for daily vehicle/IO oral gavage in every morning 9:00 am for 6 wk. Animals were restrained by tightly scruffing with the nongavage hand, and oral gavage was performed by using 1.5-in., curved, 20-gauge, stainless steel feeding needles with a 2.25 mm ball. The four groups were vehicle, IO-1× (824 mg/kg), IO-2× (1648 mg/kg), and IO-3×(2472 mg/kg) groups [19]. The vehicle group received an equivalent solution-based volume.

### 2.2. Exercise Performance Test

Exercise performance was evaluated as described previously. A low-force testing system (PicoScope 2000, Pico Technology, Cambridgeshire, UK) was used to measure the forelimb grip strength of mice in the vehicle or IO treatment groups after 6 wk and 1 h after the administration of the last treatment dose. Order to hydrolysis IO for the digestion and absorption in vivo, all exercise performance test started at 1 h after the administration IO. The amount of tensile force was measured by using a force transducer equipped with a metal bar (2 mm diameter and 7.5 cm long). A treadmill test until exhaustion was conducted from our previous study where the incline started at 10° and was progressively increased every min until exhaustion [20]. We used time to exhaustion as the main index of endurance performance. Order to reduce the amount of stress to the mice and ensure familiarization with the treadmill and swimming water, all mice were undergo to seven days of acclimatization. On day 1 animals were placed on a static treadmill and in water (length 65 cm and radius 20 cm) with 40 cm water depth maintained at 37 ± 1 °C. Subsequently, all mice set up the speed 10m/min and duration was 5 min during 3 consecutive days prior to the test on the treadmill.

### 2.3. Fatigue-Associated Biochemical Indices, Serum Biochemical Index, and Hormone Index

The effects of IO on serum lactate, ammonia, creatine kinase (CK), glucose, lactate dehydrogenase, and free fatty acid (FFA) levels were evaluated postexercise. All the mice were fasted 5 h prior to a 15 min swimming test. Prior to the test, the mice were provided with the IO supplementation; after 1 h, a 15 min unloaded swimming test commenced as described previously [21]. After a 15 min swim exercise, blood samples were immediately collected and centrifuged at 1500× *g* at 4 °C for 10 min for serum separation. The fatigue-associated biochemical indices in the serum were determined using an autoanalyzer (SYSMEX XT-2000iv, Sysmex, Kobe, Japan).

### 2.4. Tissue Glycogen Determination and Visceral Organ Weight

The stored form of glucose in the liver and muscle is glycogen, which exists mostly in liver and muscle tissues. The liver and muscle tissues were excised after the mice were euthanized and weighed for glycogen content analysis, as described previously [21].

### 2.5. Magnetic Resonance Imaging

Magnetic resonance imaging (MRI) was conducted using a 0.5T mouse MRI scanner (MesoMR, Niumag Analytical, Suzhou, China) 1 h after the intragastric administration of IO before the mice were anesthetized with 1.5% isoflurane. The respiratory rate was monitored using a pneumatic sensor to detect the depth of anesthesia during MRI acquisition, and we selected the third cross-section for MRI imaging. Subsequently, MRI was used to capture the muscle volume area.

### 2.6. Measuring Energy Metabolism and In-Cage Spontaneous Physical Activity

After 5 wk of IO supplementation, the mice were transferred to an energy-metabolism system and housed singly for 5 d. During this period, the mice were provided with free access to the food hopper and water. Respiratory gases, including water vapor, were measured with an integrated fuel-cell oxygen analyzer, spectrophotometric carbon dioxide analyzer, and capacitive water vapor partial pressure analyzer. The respiratory quotient (RQ) was calculated as the ratio of carbon dioxide production (VCO_2_) to oxygen consumption (VO_2_) [22]. Energy expenditure (EE) was calculated using the Weir equation: kcal/h = 60 × [0.003941 × VO_2_ + 0.001106 × VCO_2_] [23]. All the metabolism cages had running wheels to measure in-cage spontaneous physical activity (SPA) for 24 h over 5 d. Total wheel revolutions were recorded daily, with the total distance run per day determined by multiplying the number of wheel rotations and speed by the circumference of the wheel. This type of exercise is considered voluntary exercise [24].

### 2.7. RNA Sequencing of Muscle Tissue

Muscle tissue was obtained after the mice were euthanized, and an RNA-sequencing analysis was conducted as described previously [25,26]. After RNA extraction, purification, and library establishment, the libraries were sequenced using next generation sequencing based on the Illumina hiSeq sequencing platform. Gene counts were subsequently used as input for analysis using DESeq2 [27]. A gene ontology (GO) pathway analysis was performed using GOseq [28], and significantly expressed Kyoto Encyclopedia of Genes and Genomes (KEGG) pathways were identified using Gage [29] and visualized using Pathview.

### 2.8. Statistical Analysis

All data are expressed as mean ± standard deviation. Statistical differences between the groups were analyzed using a one-way analysis of variance, and a Cochran–Armitage test was used for dose-effect trend analysis. All statistics were performed using the PyCharm program (version 2021.2.3, Prague, Czech Republic), with *p* values <0.05 considered statistically significant.

## 3. Results

### 3.1. Effect of Six-Week IO Supplementation on General Characteristics and MRI Analysis

**Figure 1:**
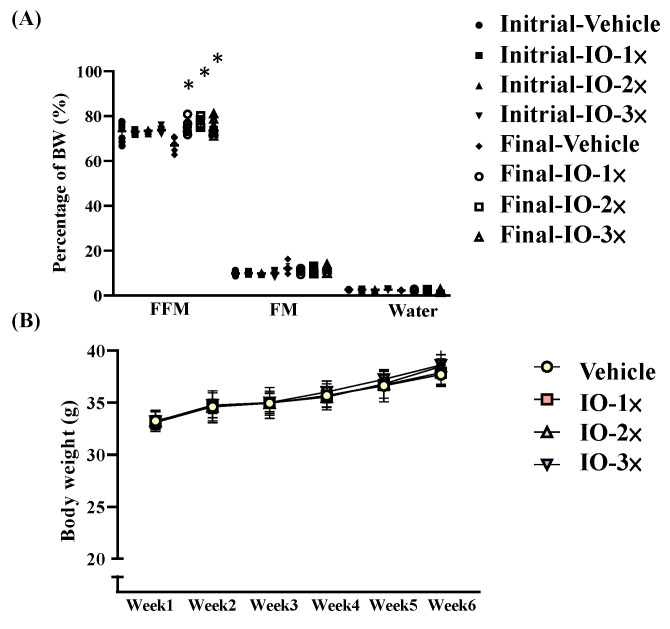
Body composition of the mice with *Inonotus obliquus* (IO) supplementation (**A**); body composition has two points of detection. The initial test was conducted before IO supplementation, and the final test was conducted 6 wk after IO supplementation (**B**). Increased body weight in IO-treated mice. Male Institute of Cancer Research (ICR) mice were supplemented with the vehicle, IO-1×, IO-2×, and IO-3× for 6 wk. Values are the mean ± standard deviation (SD) for *n* = 8 mice per group. * *p* < 0.05 compared with the vehicle group.

Table 1 lists the body weight (BW), food and water intake, and tissue weight of the mice with IO supplementation over 6 wk. These data revealed that IO supplementation led to different epididymal fat pad (EFP) and muscle weights in the IO group compared with those in the vehicle group. The body composition data were obtained using a mouse body composition analyzer (Figure 1A). The free fat mass (FFM) was significantly higher in the IO-1× (1.12-fold, *p* < 0.0001), IO-2× (1.14-fold, *p* < 0.0001), and IO-3× (1.12-fold, *p* < 0.0001) groups than in the vehicle group. No significant difference was identified in initial BW, final BW, and water intake between the groups (Figure 1B, Table 1).

**Figure 2:**
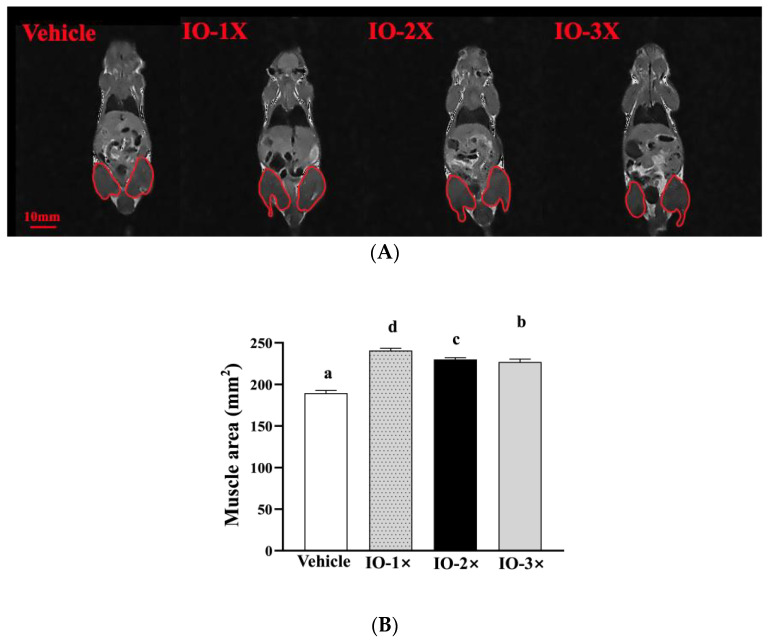
Effect of IO on the magnetic resonance imaging (**A**) of mouse muscle volume and major organ diagnostics (**B**). Different letters (a, b, c, d) indicate a significant difference at *p* < 0.05.

The MRI results are presented in Figure 2A. At wk 6, a quantitative MRI analysis of mouse muscle volume indicated a significant increase in muscular mass in the IO group compared with that in the vehicle group (Figure 2A,B). A further increase in muscle volume in the IO-1× (241 ± 2 mm^2^), IO-2× (230 ± 2 mm^2^), and IO-3× (227 ± 3 mm^2^) groups compared with that in the vehicle group (189 ± 3 mm^2^) was observed. Subsequently, muscle volume continued to increase in the IO group. We observed no abnormalities in the MRI analysis of the IO groups compared with the vehicle group after 6 wk of IO supplementation.

### 3.2. Effect of Six-Week IO Supplementation on Exercise Performance

**Figure 3:**
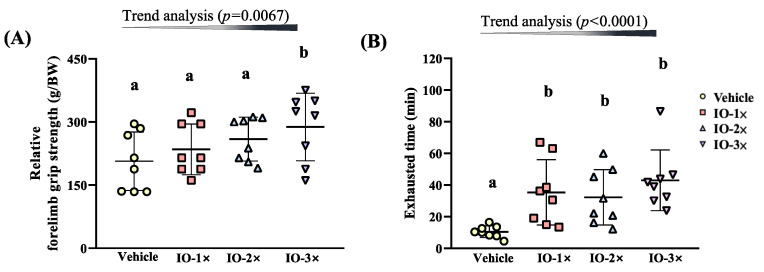
Effect of IO supplementation on exercise performance (**A**) Relative forelimb grip strength and **(B)** Exhausted treadmill test. Male ICR mice were pretreated with the vehicle, IO-1×, IO-2×, and IO-3× and underwent an exercise performance test 1 h after the final administered dose. Different letters (a, b) indicate a significant difference at *p* < 0.05.

As illustrated in Figure 3A, the grip strength of the vehicle, IO-1×, IO-2×, and IO-3× group mice was 206.9 ± 69.5, 235.0 ± 60.4, 259.4 ± 52.2, and 288.3± 80.4 g, respectively. The grip strength of the IO-3× group mice was significantly higher (1.39-fold, *p* = 0.0029) than that of the vehicle group. In the trend analysis, IO supplementation had a significant dose-dependent effect on grip strength (*p* = 0.0067).

The exhaustive treadmill test times of the vehicle, IO-1×, IO-2×, and IO-3× group mice were 10.4 ± 3.7, 35.4 ± 20.6, 32.3 ± 17.5, and 43.0 ± 19.2 min. The exhaustive treadmill times of the IO-1×, IO-2×, and IO-3× group mice were significantly higher (3.39-fold [*p* = 0.0057], 3.09-fold [*p* = 0.0141], and 4.12-fold [*p* = 0.0005], respectively) than those of the vehicle group. In the trend analysis, IO supplementation had a significant dose-dependent effect on the exhaustive treadmill time (*p* < 0.0001).

### 3.3. Effect of Six-Week IO Supplementation on the Acute Exercise Profile after a 15 Min Swimming Test

**Figure 4:**
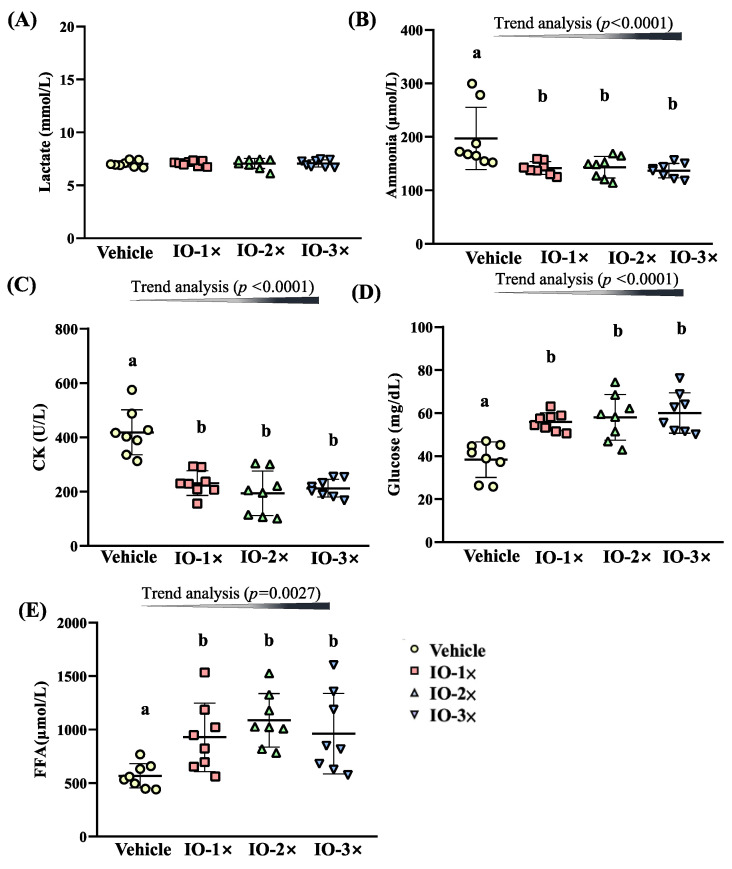
Effect of IO supplementation on the acute exercise biochemical test. Lactate (**A**); ammonia (**B**); creatine kinase (**C**); glucose (**D**); and free fatty acids (**E**). Male ICR mice were pretreated with the vehicle, IO-1×, IO-2×, and IO-3× supplementation and underwent an exercise performance test 1 h after the final administered dose. Different letters (a, b) indicate a significant difference at *p* < 0.05.

On day 43 of the supplementation period, the mice underwent a 15 min swimming test to evaluate fatigue-related biochemical indices. Serum lactate levels in the vehicle, IO-1×, IO-2×, and IO-3× groups were 7.0 ± 0.3, 7.1 ± 0.2, 7.1± 0.2, 7.1 ± 0.5, and 7.1 ± 0.3 mmol/L, with the IO groups exhibiting no significant difference compared with the vehicle group (Figure 4A). The serum ammonia levels in the vehicle, IO-1×, IO-2×, and IO-3× groups were 197 ± 58, 142 ± 12, 143 ± 20, and 137± 13 mmol/L, and the levels in the IO groups were significantly lower by 28.14% (*p* = 0.0017), 27.30% (*p* = 0.0023), and 30.52% (*p* = 0.0008), respectively, than those in the vehicle group (Figure 4B). The serum CK activity in the vehicle, IO-1×, IO-2×, and IO-3× groups were 418 ± 83, 231 ± 45, 194 ± 82, and 213 ± 33 U/L, and the activity in the IO groups were significantly lower by 44.72% (*p* < 0.0001), 53.71% (*p* < 0.0001), and 49.16% (*p* < 0.0001), respectively, than those in the vehicle group (Figure 4C). The glucose levels in the vehicle, IO-1×, IO-2×, and IO-3× groups were 38.4 ± 8.3, 55.9 ± 4.2, 58.0 ± 10.6, and 60.1± 9.4 mg/dL, and the levels in the IO groups were significantly higher (1.46-fold [*p* = 0.0003], 1.51-fold [*p* < 0.0001], and 1.57-fold [*p* < 0.0001]) than those in the vehicle group (Figure 4D). The FFA levels were 567 ± 113, 928 ± 320, 1087 ± 250, and 962 ± 376 µmol/L in the vehicle, IO-1×, IO-2×, and IO-3× groups, respectively. The FFA levels in the IO-1×, IO-2×, and IO-3× groups were significantly higher (1.64-fold [*p* = 0.0165], 1.91-fold [*p* = 0.0010], and 1.70-fold [*p* = 0.0093], respectively) than those in the vehicle group (Figure 4E). Furthermore, according to the trend analysis, the serum ammonia (*p* < 0.0001), CK (*p* < 0.0001), glucose (*p* < 0.0001), and FFA (*p* = 0.0027) levels were significantly dose dependent. Thus, 6-wk IO supplementation affected ammonia, CK, glucose, and FFA levels, reducing the fatigue biochemical indices.

### 3.4. Effect of Six-Week IO Supplementation on Glycogen Content

**Figure 5:**
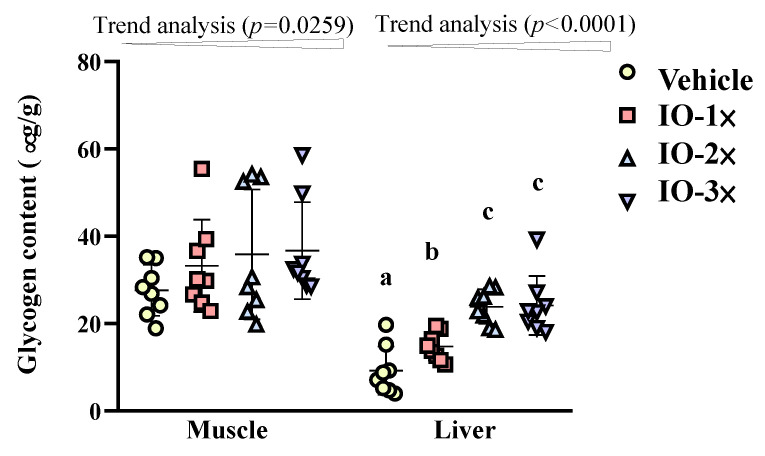
Effect of IO on muscle and liver glycogen levels at rest. All the mice were euthanized and tested for glycogen levels in muscle tissues 1 h after the final treatment. Data represent the mean ± SD of eight mice in each group. A one-way analysis of variance (ANOVA) was used for the analysis. Different letters (a, b, c) indicate a significant difference at *p* < 0.05.

As depicted in Figure 5, the muscle glycogen of the vehicle IO-1×, IO-2×, and IO-3× groups was 27.6 ± 5.8, 33.2 ± 10.6, 35.9 ± 14.9, and 36.7 ± 11.1 µg/g, respectively. Although no significant difference was identified between the IO groups and the vehicle group in terms of muscle glycogen, the trend analysis demonstrated a significant dose dependence when IO supplementation was increased (*p* = 0.0259). The liver glycogen content of the vehicle, IO-1×, IO-2×, and IO-3× groups was 9.3 ± 5.5, 14.8 ± 3.2, 23.9 ± 3.9, and 24.2 ± 6.7 µg/g, respectively. The liver glycogen content of the IO-1×, IO-2×, and IO-3× groups was significantly higher (1.60-fold [*p* = 0.0368], 2.58-fold [*p* < 0.0001], and 2.61-fold [*p* < 0.0001], respectively) than that of the vehicle group.

### 3.5. Effect of Six-Week IO Supplementation on Energy Metabolism

**Figure 6:**
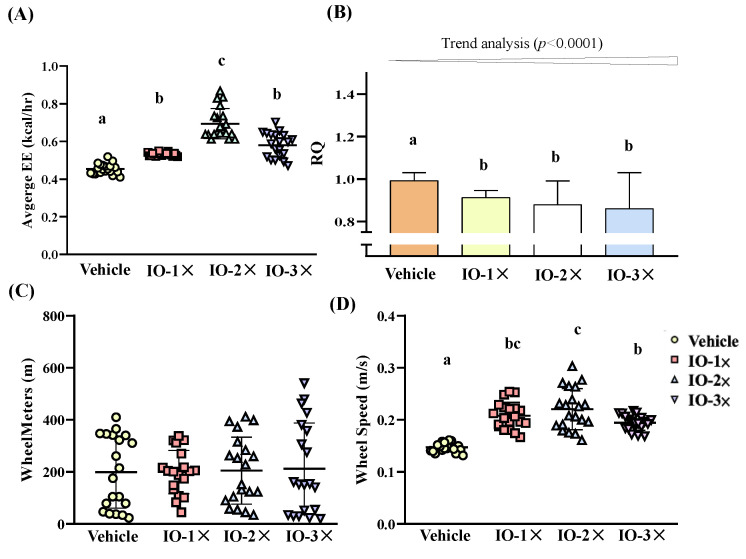
Effect of IO supplementation on energy metabolism and in-cage spontaneous physical activity (**A**) Average energy expenditure, (**B**) Respiratory quotient, (**C**) Distance of spontaneous exercise, (**D**) Wheel speed of spontaneous exercise. Mice were pretreated with the vehicle, IO-1×, IO-2×, and IO-3× for 6 wk. They were measured using respirometric indirect calorimetry over 72 h during the IO or vehicle infusions. Data represent the mean ± SD of eight mice in each group. One-way ANOVA was used for the analysis. Different letters (a, b, c) indicate a significant difference at *p* < 0.05.

We measured energy expenditure (EE) through respirometric indirect calorimetry over 72 h during IO or vehicle infusions. We assessed the effect of IO on EE by measuring VCO_2_ and VO_2_ to calculate RQ, which is used to estimate the type of fuel utilized [30]. The average EE in the vehicle, IO-1×, IO-2×, and IO-3× groups was 0.454 ± 0.028, 0.532 ± 0.009, 0.694 ± 0.081, and 0.580 ± 0.066 kcal/h, respectively (Figure 6A). The average EE of the IO-1×, IO-2×, and IO-3× groups was significantly higher (1.17-fold [*p* < 0.0001], 1.53-fold [*p* < 0.0001], and 1.28-fold [*p* < 0.0001], respectively) than that of the vehicle group. The RQ of the vehicle, IO-1×, IO-2×, and IO-3× groups ice was 0.99 ± 0.04, 0.91 ± 0.03, 0.88 ± 0.11, and 0.86 ± 0.17 VCO_2_/VO_2_, respectively (Figure 6B). The IO-1×, IO-2×, and IO-3× RQ index was significantly decreased by 8.0% (*p* < 0.0191), 11.4% (*p* < 0.0001), and 13.3% (*p* < 0.0001), respectively, compared with that of the vehicle group. In the trend analysis, the RQ index exhibited a significant decrease (*p* < 0.0001) following IO supplementation. We also analyzed in-cage SPA. Although the total wheel meters were not significant in any group (Figure 6C), the wheel speed in the IO-1×, IO-2×, and IO-3× groups was significantly higher (1.41-fold [*p* < 0.0001], 1.50-fold [*p* < 0.0001], and 1.32-fold [*p* < 0.0001], respectively) than that of the vehicle group (Figure 6D). Thus, although IO treatment had little impact on the total meters in SPA, it enhanced exercise performance (increased wheel speed), suggesting that the supplementation increased EE through exercise performance. The increase in the FFM content was considerably affected by the IO-induced increase in the average EE [31].

### 3.6. Effect of Six-Week IO Supplementation on Biochemical Variables

The levels of alanine aminotransferase, aspartate aminotransferase, total protein, creatinine, urea assay, and glucose were nonsignificant in each group. Albumin levels and the lipid profile (total cholesterol [TC], triacyl glycerol [TG]) were significantly lower in the IO group than in the vehicle group. In addition, IO supplementation resulted in a dose-dependent decrease in TC and TG levels (Table 2). 

### 3.7. Effect of Six-Week IO Supplementation on the Gene Ontology Analysis and Enrichment Analysis of the Kyoto Encyclopedia of Genes and Genomes in Muscle

**Figure 7:**
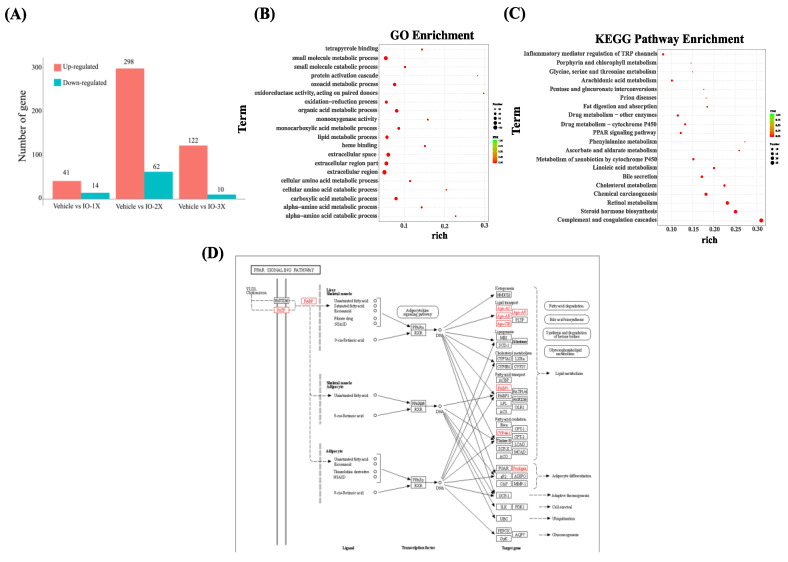
IO supplementation of gene expression analysis in mouse muscle. (**A**) Number of differentially expressed genes in the vehicle and IO mouse groups at significant expression levels in muscle. (**B**) Gene ontology analysis of the top 20 most enriched pathways following IO treatment based on target genes. (**C**) KEGG pathway enrichment of IO treatment. Top 20 pathways enriched based on target genes (the abscissa is the enrichment factor, the ordinate is the pathway name, the size of the dot indicates the number of target genes, and the color represents the *p*-value). (**D**) Analysis of the PPAR signaling pathway. Red represents the targets of the IO treatment in the upregulation of the PPAR pathway.

To explore the beneficial effect of IO supplementation, we analyzed the action mechanism in the vehicle- and IO-supplemented mice using transcriptome sequencing of the mouse muscle. Figure 7A presents the analysis of gene expression in the IO and vehicle groups. The DEseq R package was used to analyze the differences in gene expression, and the screening conditions are expressed as the expression difference multiple |log2foldchange| > 1, with a significant difference of *p* < 0.05. The vehicle versus IO-2× result demonstrated the largest amount of gene upregulation compared with vehicle versus IO-1× and vehicle versus IO-3×. In the IO-2× group, 298 genes were upregulated and 62 genes were downregulated. We then constructed a Venn diagram to obtain the 16 key target genes for the IO supplementation group (Supplementary Table S1). To explore the mechanism underlying the IO effect on improved exercise performance and changes in body composition, the target genes were imported into the DAVID database for the GO enrichment (Figure 7B) and biological pathway analysis of KEGG (Figure 7C). As presented in Figure 7B, with regard to the top 20 significantly upregulated genes from the GO enrichment analysis, IO treatment had the lowest false discovery rate value for lipid oxidation activity, monocarboxylic acid metabolic process, small molecule metabolic process, extracellular region, oxoacid metabolic process, carboxylic acid metabolic process, and organic acid metabolic process. The top 20 pathways involved in IO treatment with the most significant expression were the PPAR signaling pathway, phenylalanine metabolism, ascorbate and aldarate metabolism, cholesterol metabolism, chemical carcinogenesis, and steroid hormone biosynthesis (Figure 7C). We used the target genes and their corresponding effective components to analyze the effect of IO treatment on PPAR signaling pathways (Figure 7D). Red represents the upregulated pathways after IO treatment. The results demonstrated that fatty acid transport proteins, fatty acid binding protein (FABP), apolipoprotein (APO)-AⅠ, APO-AⅡ, APO-CⅢ, APO-AV, FABP1, cytochrome P450 (CYP4A1), and perilipin are the key nodes in the network. The lipid transport pathway has the most upregulated genes after IO treatment.

## 4. Discussion

The results demonstrated that 6-wk IO supplementation increased muscle volume and decreased fat weight (EFP tissue) in the mice. IO supplementation enhanced mouse exercise performance through energy expenditure (Figure 2) by increase glucose content during exercise. Studies also revealed that IO increases the swimming time and the glycogen content of the liver and muscle, that is the important energy fuel source during exercise [11]. In other studies, the IO polysaccharide increased GRAF1 expression in mouse gastrocnemius muscles [32], which maintain muscle membrane integrity and muscle repair [33]. According to our results, IO may influence cross-talk between muscle and lipid metabolism to improve glucose regulation. The lipid tissue alleviate (Figure 2) and increase the glucose uptake could clearly found the glycogen levels and FFA content were increased by 6-wk IO treatment (Figure 5). Therefore, a recent study suggested that elevated FFA immediately after exercise can provide an energy source for skeletal muscles, restoring glycogen concentrations [25]. In previous study reveal that cholesterol-rich (similar with IO) food can restore liver glycogen content to enhance exercise time [34] was the other evidence that IO has potential conditioning the glucose level absorption and increase insulin sensitive which is relative with exercise performance.

In addition, we identified CK as the metabolic biomarker after acute exercise. CK levels were reduced after acute exercise in the IO treatment group; thus, CK is deemed to play a key role during strenuous exercise with muscle fatigue [35]. Numerous studies have focused on IO for treating diabetes [7,19,36]. Our results suggest that IO could affect insulin secretion and glycemic control [37], which were adjusted after exercise, causing fatigue. Previous studies have also reported that IO treatment increases insulin-stimulated glucose uptake [9]. The increase in FFA may alter hepatic lipid storage and/or insulin resistance to some extent, supporting blood glucose increase after exercise [38,39]. Consequently, IO treatment alters skeletal muscle insulin resistance to reduce acute exercise metabolic waste. Our results demonstrated that IO treatment for 6 wk increased the effects of exercise performance on glucose and lipid metabolism rather than moderating acute exercise, and this may, in part, explain improved exercise performance. To examine how IO affects body composition and exercise performance, we analyzed data related to activated PPAR, a lipid-activated transport factor that regulates APO-AI, APO-AII, and APO-CIII gene expression. According to Szychowski et al. [40], IO extract consist of the triterpenoids, steroids fractions and polysaccharide fractions and those substances are the key regulatory substances to activate PPARs.

The mice treated with IO became exhausted later than those in the vehicle group and exhibited higher rates of glycogen depletion in the liver than in skeletal muscles. According to metabolic data, IO supplementation increased EE and reduced the RQ value, indicating an increase in fat oxidation and a decrease in carbohydrate oxidation [40]. These data indicate that during 6-wk IO treatment, the mechanisms underlying improved exercise performance involved increased glycogen content [41] and the rapid repletion of glycogen stores, both of which maximize lipid oxidation and oxidative capacity for the performance recovery of endurance athletes [42]. Although EE increased during IO treatment, in our study, BW exhibited no difference between the groups; changes in EE and fat oxidation in weight change are contentious [43]. IO may be used to treat obesity and may be able to modulate fat oxidation through nutrients and drugs used for treating diabetes.

In addition, the present study revealed that the increases in the expression of lipid-activated transport factor genes in energy balance pathways and body composition changes resulting from IO treatment are similar to responses to exercise training programs [44]. The mice in the IO-2X groups increased their SPA exercise performance through enhanced wheel speed; thus, IO could be used to increase lipid oxidation as a predominant energy substrate [45].

In the present study, IO supplementation decreased the lipid levels in mice. This can be attributed to an increase in APO-A1 expression in the IO treatment group. The *APO-A1* gene in the high-density lipoprotein pathway is associated with muscle differentiation [10]. IO supplementation activated the PPAR pathway, inducing gene expression associated with glucose uptake, fatty acid synthesis, and lipid storage [46]. We investigated the glycogen level in the muscle and liver. Liver glycogen levels increased glucose storage through IO treatment. Furthermore, the increase in glycogen storage was consistent with an increase in the mouse exercise exhaustion time, reduced fat accumulation, and enhanced FFA after acute exercise following IO treatment. This observation suggests that the activation of the PPAR pathway regulates lipid catabolism in skeletal muscles.

The dose-dependent analysis, some IO-3X data were not effective than IO-1X and IO-2X, we infer that IO show no significant benefit in this increased dosage to 3X. Thus, IO-2X was the acceptable dose as nutraceuticals.

Notably, this increase in gene expression in the PPAR pathway is mirrored by increased improve insulin sensitivity in skeletal muscle, our data showed that increase of absolute and relative muscle mass. The increase of muscle mass could lead to greater mitochondria content and respiration which ultimately facilitates the increase of VO_2_ and hence endurance performance when assessed by time to exhaustion. Therefore, IO may counteract skeletal muscle fatigue to extend exercise time. There may be some possible limitations in this study. Our study was only IO supplementation without training-related procedures. As all mice were not having any exercise training during supplementation period, we therefore do not know the combination of exercise training and IO supplementation can further increase the endurance performance when compare with IO supplementation along. We did not compare moderate (6 weeks) vs. prolonged supplementation (12 week) on muscle and endurance performance in mice. However, this has not been investigated by any current studies to date and thus warrants further investigation.

## 5. Conclusions

In this study, 6-wk IO supplementation significantly decreased the EPF weight and had beneficial effects on muscle content. Exercise performance was significantly improved in the IO group through increased glucose and FFA content after acute exercise. The glycogen storage ability also increased. The results of RNA sequencing revealed that FATP, FABP, APO-AI, APO-AII, APO-CIII, APO-AV, FABP1, CYP4A1, and perilipin expression increased in the network. The lipid transport pathway was the most upregulated during IO treatment. These data indicate that IO increases lipid transport and has positive effects on glucose uptake during exercise. Further studies to understand the up-regulated. To solely isolate the effect of IO supplementation on lipid transportation, further studies should focus on IO supplementation with and without resistance exercise so that the separate and the combined effect of IO supplementation can be truly revealed. However, this is unrelated to the main purpose of this study and thus warrants further investigation.

## Figures and Tables

**Table 1:** General characteristics of mice with IO supplementation.

Characteristic	Vehicle	IO-1×	IO-2×	IO-3×	Trend Analysis
Initial BW (g)	33.25 ± 11.8	33.10 ± 11.7	33.28 ± 1.8	33.20 ± 11.7	0.8641
Final BW (g)	37.69 ± 13.3	37.89 ± 13.4	38.49 ± 13.6	38.63 ± 13.7	0.0968
Food intake (g/day)	4.99 ± 1.8	4.73 ± 1.7	4.84 ± 1.7	4.82 ± 1.7	0.5274
Water intake (g/day)	6.99 ± 2.5	6.81 ± 2.4	7.25 ± 2.6	7.04 ± 2.5	0.1163
Liver (g)	1.09 ± 0.4	1.03 ± 0.4	1.03 ± 0.4	1.03 ± 0.4	0.0848
Lung (g)	0.22 ± 0.1	0.21 ± 0.1	0.21 ± 0.1	0.21 ± 0.1	0.0699
Heart (g)	0.18 ± 0.1	0.18 ± 0.1	0.19 ± 0.1	0.19 ± 0.1	0.7387
EFP (g)	0.69 ± 0.2 ^a^	0.63 ± 0.2 ^ab^	0.57 ± 0.2 ^bc^	0.54 ± 0.2 ^c^	<0.0001
Muscle (g)	0.40 ± 0.1 ^a^	0.44 ± 0.2 ^bc^	0.45 ± 0.2 ^c^	0.42 ± 0.1 ^ab^	0.5613
BAT (g)	0.17 ± 0.1 ^a^	0.21 ± 0.1 ^b^	0.20 ± 0.1 ^ab^	0.20 ± 0.1 ^ab^	0.4940
Relative Liver weight (%)	2.89 ± 1.0	2.72 ± 1.0	2.68 ± 0.9	2.66 ± 0.9	0.0159
Relative Lung weight (%)	6.77 ± 2.4	7.87 ± 2.8	7.81 ± 2.8	7.79 ± 2.8	0.4397
Relative Heart weight (%)	2.20 ± 0.8	2.30 ± 0.8	2.46 ± 0.9	2.39 ± 0.8	0.7175
Relative EFP weight (%)	3.91 ± 1.4 ^a^	3.31 ± 1.2 ^ab^	3.14 ± 1.1 ^ab^	3.10 ± 1.1 ^b^	0.0479
Relative Muscle weight (%)	14.84 ± 5.2 ^a^	19.41 ± 6.9 ^b^	18.98 ± 6.7 ^b^	17.57 ± 6.2 ^ab^	0.5518
Relative BAT weight (%)	3.51 ± 1.2 ^a^	6.39 ± 2.3 ^b^	6.57 ± 2.3 ^b^	6.48 ± 2.3 ^b^	0.0021

Data are presented as mean ± SD, *n* = 8 mice/group. Different letters (^a,b,c^) in the same row indicate a significant difference at *p* < 0.05. Muscle mass includes both gastrocnemius and soleus muscles at the back of the lower legs. BW, body weight; BAT, brown adipose tissue; EFP, epididymal fat pad. The mice were supplemented for 6 wk with the vehicle (glucose water), IO-1× (824 mg/kg/day IO), IO-2× (1648 mg/kg/day IO), or IO-3× (2472 mg/kg/day IO).

**Table 2:** Effect of 6-week IO supplementation on biochemical variables.

Parameter	Vehicle	IO-1×	IO-2×	IO-3×	Trend Analysis
ALT (U/L)	50.9 ± 18	51.3 ± 18.1	43.1 ± 15.2	47.3 ± 13.9	0.2698
AST (U/L)	114.8 ± 40.6	100.1 ± 35.4	99.9 ± 35.3	116.9 ± 41.3	0.6901
TP (G/L)	55.4 ± 19.6	57.6 ± 20.4	56.8 ± 20.1	57.6 ± 20.4	0.1849
ALB (G/L)	41.3 ± 14.6 ^a^	41.3 ± 14.6 ^a^	40.2 ± 14.2 ^ab^	39.1 ± 13.8 ^b^	0.0021
BUN (mmol/L)	8.3 ± 2.9	8.1 ± 2.9	8.2 ± 2.9	7.8 ± 2.8	0.1667
CRE (μmol/L)	39.6 ± 14	44.9 ± 15.9	48.6 ± 17.2	51.4 ± 18.2	<0.0001
UA (μmol/L)	121.8 ± 43.1	115.5 ± 40.8	131.1 ± 46.4	122.6 ± 43.3	0.5636
TC (mg/dL)	179.2 ± 63.4 ^a^	136.2 ± 48.2 ^b^	131.1 ± 46.3 ^b^	130.4 ± 46.1 ^b^	<0.0001
TG (mg/dL)	137.8 ± 48.7 ^a^	97.6 ± 34.5 ^b^	85.1 ± 30.1 ^b^	81.2 ± 28.7 ^b^	<0.0001
Glucose (mg/dL)	82.9 ± 29.3	81 ± 28.7	81.5 ± 28.8	85.7 ± 30.3	0.6562

Data are expressed as mean ± SD, *n* = 8 mice/group. Different letters (^a, b^) in the same row indicate significant differences at *p* < 0.05 using one-way ANOVA. AST, aspartate aminotransferase; ALT, alanine amino transferase; TP, total protein; ALB, albumin; BUN, blood urea nitrogen; CRE, creatinine; UA, urea assay; TC, total cholesterol; TG, triacyl glycerol.

## Data Availability

Not applicable.

## Supplementary Materials

The following supporting information can be downloaded at: https://www.mdpi.com/article/10.3390/nu14235007/s1, Table S1. Effect of 6-week IO supplementation on up-regulated 16 key target genes.
