# Supplementary material for: Effect of Inonotus obliquus Extract Supplementation on Endurance Exercise and Energy-Consuming Processes through Lipid Transport in Mice
Source: Nutrients. 2022 Nov 25;14(23):5007. doi: 10.3390/nu14235007 (PMC9737630; doi:10.3390/nu14235007)
Supplement: Supplementary file 1 [file nutrients-14-05007-s001.zip › nutrients-1996995-supplementary.pdf]

Table S1. Effect of 6-week IO supplementation on up-regulated 16 key target genes

Ensembl ID	Symbol	Description	log_2FoldChange	*P*-value
ENSMUSG00000029273	Sult1d1	Enables aryl sulfotransferase activity	Inf	0.0168
ENSMUSG00000026368	F13b	This gene encodes subunit B of the coagulation factor XIII that catalyzes the final step of the blood coagulation pathway	Inf	0.0127
ENSMUSG00000032079	Apoa5	Predicted to enable several functions, including cholesterol binding activity	Inf	0.0013
ENSMUSG00000060613	Cyp2c70	Predicted to enable heme binding activity and monooxygenase activity	Inf	0.0014
ENSMUSG00000109764	Klkb1	This gene encodes a member of the kallikrein subfamily of serine proteases that are involved in diverse physiological functions such as skin desquamation, tooth enamel formation, seminal liquefaction, synaptic neural plasticity and brain function	Inf	0.0308
ENSMUSG00000064246	Chil1	Predicted to enable chitin binding activity	1.344144406	0.0230
ENSMUSG00000020051	Pah	Enables phenylalanine 4-monooxygenase activity	7.820402799	0.0088
ENSMUSG00000079015	Serpina1c	Predicted to enable identical protein binding activity; protease binding activity; and serine-type endopeptidase inhibitor activity	6.384979074	0.0277
ENSMUSG00000029260	Ugt2b34	Predicted to enable UDP-glycosyltransferase activity	Inf	0.0050
ENSMUSG00000034528	Hsd17b13	Predicted to enable oxidoreductase activity, acting on the CH-OH group of donors, NAD or NADP as acceptor and steroid dehydrogenase activity	Inf	0.0026
ENSMUSG00000023070	Rgn	Enables gluconolactonase activity	8.639440534	0.0209
ENSMUSG00000022181	C6	Involved in positive regulation of activation of membrane attack complex and positive regulation of angiogenesis	6.345014096	0.0188
ENSMUSG00000054422	Fabp1	Enables chromatin binding activity	7.078111575	0.0056
ENSMUSG00000067225	Cyp2c54	Enables arachidonic acid epoxygenase activity and linoleic acid epoxygenase activity	Inf	0.0089
ENSMUSG00000041698	Slco1a1	Enables organic anion transmembrane transporter activity	8.361152802	0.0183
ENSMUSG00000103476	Strit1	Enables enzyme activator activity	2.074906743	0.0068
